# NMR solution structure and function of the C-terminal domain of eukaryotic class 1 polypeptide chain release factor

**DOI:** 10.1111/j.1742-4658.2010.07672.x

**Published:** 2010-06

**Authors:** Alexey B Mantsyzov, Elena V Ivanova, Berry Birdsall, Elena Z Alkalaeva, Polina N Kryuchkova, Geoff Kelly, Ludmila Y Frolova, Vladimir I Polshakov

**Affiliations:** 1 Center for Magnetic Tomography and Spectroscopy, M. V. Lomonosov Moscow State UniversityRussia; 2 Engelhardt Institute of Molecular Biology, Russian Academy of SciencesMoscow, Russia; 3 Division of Molecular Structure, MRC National Institute for Medical ResearchLondon, UK; 4 Chemical Department, M. V. Lomonosov Moscow State UniversityRussia; 5 MRC Biomedical NMR CentreNIMR, London, UK

**Keywords:** human eukaryotic class 1 polypeptide chain release factor (eRF1), NMR structure and dynamics, stop codon recognition specificity, termination of protein synthesis

## Abstract

Termination of translation in eukaryotes is triggered by two polypeptide chain release factors, eukaryotic class 1 polypeptide chain release factor (eRF1) and eukaryotic class 2 polypeptide chain release factor 3. eRF1 is a three-domain protein that interacts with eukaryotic class 2 polypeptide chain release factor 3 via its C-terminal domain (C-domain). The high-resolution NMR structure of the human C-domain (residues 277–437) has been determined in solution. The overall fold and the structure of the β-strand core of the protein in solution are similar to those found in the crystal structure. The structure of the minidomain (residues 329–372), which was ill-defined in the crystal structure, has been determined in solution. The protein backbone dynamics, studied using ^15^N-relaxation experiments, showed that the C-terminal tail 414–437 and the minidomain are the most flexible parts of the human C-domain. The minidomain exists in solution in two conformational states, slowly interconverting on the NMR timescale. Superposition of this NMR solution structure of the human C-domain onto the available crystal structure of full-length human eRF1 shows that the minidomain is close to the stop codon-recognizing N-terminal domain. Mutations in the tip of the minidomain were found to affect the stop codon specificity of the factor. The results provide new insights into the possible role of the C-domain in the process of translation termination.

## Introduction

Termination of translation in eukaryotes is governed by the cooperative action of two interacting polypeptide chain factors, eukaryotic class 1 polypeptide chain release factor (eRF1) and eukaryotic class 2 polypeptide chain release factor 3 (eRF3). The major functions of eRF1 include recognition of each of the three stop codons (UAA, UAG, or UGA) in the decoding center of the small ribosomal subunit and the subsequent peptidyl-tRNA hydrolysis. eRF3 is a ribosome-dependent and eRF1-dependent GTPase encoded by an essential gene that enhances the termination efficiency by stimulating the activity of eRF1 [1–4].

eRF1 contains three structurally separated domains, each of which can be assigned a specific function. The N-terminal domain (N-domain) is involved in the recognition of the stop codon [1,5,6]. The middle domain (M-domain) catalyzes the hydrolysis of the peptidyl-tRNA ester bond within the peptidyltransferase center of the 60S ribosome subunit [7,8]. The C-terminal domain (C-domain) binds to eRF3 [9–12], and this interaction increases the efficiency of translation termination [13,14]. However, in a simplified *in vitro* assay for the measurement of release factor (RF) activity, eRF1, deprived of the C-domain, still retains its RF activity [15]. The combination of the human M-domain and C-domain, in the absence of the N-domain, is able to bind to the mammalian ribosome and to induce the GTPase activity of eRF3 [16].

It has been found that eRF1 and eRF3 form ternary and quaternary complexes in solution with GTP and Mg^2+^ (eRF1–eRF3–GTP and eRF1–eRF3–GTP–Mg^2+^) [17]. Yeast two-hybrid and deletion analyses have revealed that residues 281–305 and 411–415 of human eRF1 are important for its binding to eRF3, but the last 22 residues (415–437) are not significant for this process [11]. In contrast, in the case of eRF1s from the budding and fission yeast, the last 19 residues of the C-terminal fragment are necessary for the eRF1–eRF3 interaction [9,12]. As residues 300–303 and 411–412 correspond to the β-sheets in the central hydrophobic core of the C-domain, it might be expected that truncation of these residues would lead to destabilization of the whole structure. This suggestion is in full agreement with recent studies on the yeast Y410S C-domain mutant [18].

The structure, dynamics and functions of the C-domain have been studied much less intensively than those of the M-domain or the N-domain. In the currently available crystal structure of human eRF1 [19], coordinates exist only for the atoms that belong to the main rigid core of the C-domain, and consequently the C-domain structure has extensive unresolved fragments in its mobile regions. More recently [20], the crystal structure of human eRF1 in a complex with the truncated form of eRF3 (residues 467–662) has been solved. In particular, it has been found that the two α-helices, α8 and α11, which belong to the main rigid core of the C-domain, together with Arg192 and Arg203 of the M-domain [21], form the interface with eRF3. However, all of the mobile regions that could not be seen in the crystal structure of human eRF1 [19] still remained undetermined in the structure of the eRF1–eRF3 complex [20].

We report here the high-resolution NMR structure of the human C-domain in solution, and present data on its dynamics. On the basis of the structural data, we have performed a mutational analysis of the C-domain and investigated the impact of the mutants on stop codon recognition.

## Results

### Resonance assignment

^1^H, ^13^C and ^15^N chemical shifts were made for 99% of the protein backbone resonances of the isolated C-domain. Only Asn277, Asn325, and Gln397, whose amide group HN and ^15^N signals could not be reliably determined because of signal overlap problems, were not assigned. More than 78% of all of the observed side chain ^1^H, ^13^C and ^15^N chemical shifts were also determined.

**Fig. 1:**
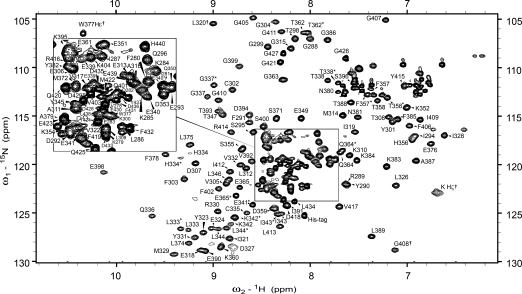
^1^H,^15^N-HSQC spectrum of the C-domain. Amide signals from residues that belong to the open protein conformation are marked with asterisks. †marked peaks correspond to folded resonances, which would otherwise appear outside the spectral region shown.

The ^1^H,^15^N-heteronuclear single quantum coherence (HSQC) spectra, measured over the temperature range 288–313 K, showed only a minor effect of temperature on the existence and line widths of the protein backbone resonances. This suggests the absence of multiple conformations that interconvert on the millisecond time scale. However, for several residues situated between positions 329 and 372 (in particular, residues 333–344, 351, and 357–370) a duplicated set of signals of approximately equal intensity was observed (Fig. 1; Fig. S1). This clearly indicates the presence of two conformational states of residues 329–372 (minidomain) of eRF1, which is highly enriched in polar and charged residues.

Refolding of the C-domain leads to the presence of only one conformational state. The refolding was carried out by lowering the pH of the protein solution from 7.0 to 3.5, and then restoring the pH to its initial value. It is also worth noting that the relative populations of the two conformational states are affected by the components of different diluted liquid crystalline media. For example, in a solution of lipid bicelles [1,2-dihexanoyl-*sn*-glycero-3-phosphocholine (DHPC)/1,2-dimyristoyl-*sn*-glycero-3-phosphocholine (DMPC)] [22], a set of signals is observed that belongs to one conformation of the minidomain, whereas in the poly(ethylene glycol)-based system [23], another set of signals could be detected. Therefore, the sizes of the relative populations and possibly the rate of conformer interconversion are sensitive to the environment of the domain.

**Fig. 2:**
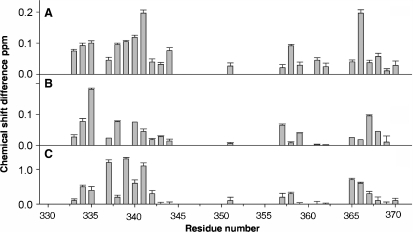
Protein backbone chemical shift differences between the resonances from the two conformations of loop 357–367. Absolute values of chemical shift differences are shown for: (A) Hα resonances; (B) HN signals; and (C) amide ^15^N resonances.

For the great majority of the residues in the minidomain, the differences between the chemical shifts of the two conformational states are sufficiently large to allow sequential assignments based on the use of ^1^H,^13^C,^15^N triple-resonance experiments (3D experiment correlating the amide HN and the Cα signals, 3D experiment correlating the amide HN and the Cα signal of the preceding amino acid, 3D experiment correlating the amide NH with the Cα and Cβ signals, 3D experiment correlating the amide NH with the Cα and Cβ signals of the preceding amino acid, and 3D experiment correlating the amide NH with the C′ signal of the preceding amino acid). Figure 2 presents the distribution of the chemical shift differences between the two protein conformers for the backbone amide proton, nitrogen and Hα signals for the minidomain. These differences are concentrated in regions 333–344 and 357–370, presumably reflecting differences in the structures in these regions. It should be noted that there are no detectable differences in chemical shifts for the remaining residues.

### Structure determination

**Fig. 3:**
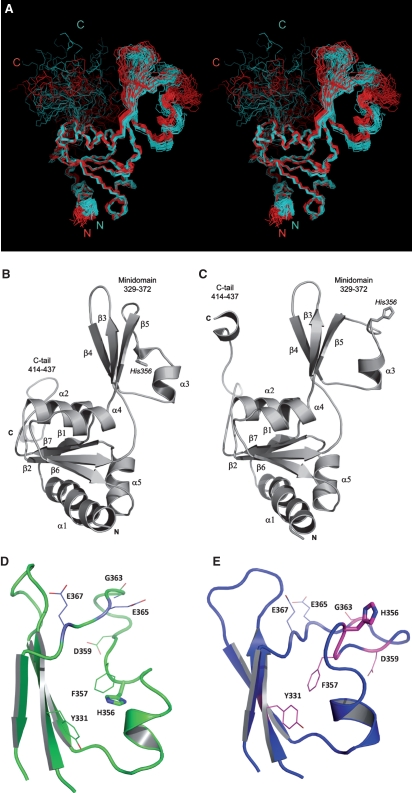
The solution structure of the C-domain. (A) Stereo view of the ensemble of the final 48 calculated structures. Twenty-four structures of the closed protein conformer are shown in red, and 24 structures of the open conformer are shown in cyan. The N-termini and C-termini are labeled. (B, C) The topology of the secondary structure elements of the open (B) and closed (C) protein conformers. (D, E) The conformations of the minidomain in the open (D) and closed (E) protein conformers. The residues participating in key interactions that could stabilize the two conformers of the minidomain are highlighted.

The existence of two distinct sets of resonances for the minidomain allowed the determination of two families representing the two conformational states of the solution structure of the C-domain (shown as a stereo view in Fig. 3A). The structure determination was based on more than 2140 experimental restraints, using data obtained at 288 and 313 K (Table 1). This work made use of the standard double-resonance ^15^N,^1^H-NMR and triple-resonance ^15^N,^13^C,^1^H-NMR experiments applied to ^13^C-labeled and/or ^13^N-labeled samples of the human C-domain. For most of the protein residues, the number of NOE restraints per residue is between 15 and 25 (Fig. S2). However, the C-terminus and fragment 336–338 have significantly lower numbers of measured distance restraints. Therefore, extensive use of residual dipolar couplings (RDCs), measured in several alignment media, was important for the determination of the structures of the conformers of the C-domain. The dipolar couplings provided long-distance information on the global folding of both conformers.

**Table 1:** Statistics for the two ensembles of the calculated structures of the human C-domain (24 structures for the open conformer and 24 for the closed conformer were analyzed).

	Open	Closed
Restraints used in the structure calculation		
Total NOEs	1857	1852
Long range (|*i*–*j* | > 4)	497	490
Medium range (1 < |*i*–*j* | ≤ 4)	332	332
Sequential (|*i*–*j* | = 1)	516	516
Intraresidue	512	514
Residue dipolar couplings, ^1^D_NH_	90	69
Dihedral angles, total	216	214
Phi (*ϕ*)	108	107
Psi (*ψ*)	108	107
Restraint violations and structural statistics (for 24 structures)		
No NOE and dihedral angle violations over 0.2 Å and 5°, respectively		
Average rmsd over ensemble		
From experimental restraints		
Distance (Å)	0.017 ± 0.001	0.019 ± 0.004
Dihedral angles (°)	0.42 ± 0.06	0.4 ± 0.1
From idealized geometry		
Bonds (Å)	0.0022 ± 0.0001	0.0025 ± 0.0005
Bond angles (°)	0.43 ± 0.01	0.49 ± 0.09
Improper angles (°)	0.34 ± 0.01	0.40 ± 0.08
Percentage of residues in the most favorable region of the Ramachandran map	91.1	85.5
Percentage of residues in disallowed region of the Ramachandran map	0	0
Superimposition of the structures on the representative structure		
rmsd over backbone C, CA, O and N atoms of residues 277–328 and 373–413 (Å) of the hydrophobic core	0.42	0.42

The structure of the protein core (residues 277–328 and 373–413) in both conformers (Fig. 3B,C) is in good agreement with that of the corresponding part of the crystal structure [19]. Four β-strands (β1, 301–303; β2, 320–323; β6, 389–392; and β7, 409–412) form a β-sheet with three antiparallel strands (1, 2 and 7) and strand 6, which is parallel to strand 2. β-Strands are located between the four α-helices (α1, 278–294; α2, 305–313; α4, 374–381; and α5, 397–405), with two of the α-helices on one side of the β-sheet and two on the other side. The rmsd of the heavy atoms (Cα, C, and N) of the protein core, when the NMR structures of both conformers are superimposed on the crystal structure of human eRF1, is 1.58 ± 0.06 Å.

The fold of the minidomain, for both protein conformations, contains identical secondary structural elements: β-strands (β3, 329–335; β4, 339–344; and β5, 367–372) and a distorted α-helix (α3, 348–356) (Fig. 3B,C). The three β-strands of the minidomain are all antiparallel, and form a single β-structure.

### Two protein conformers

The main structural difference between the two protein conformers is in the orientation of α3 (residues 348–356), with respect to the β-structure of the minidomain and the corresponding tilt of the loop (residues 357–367) between α3 and β5 (Fig. 3B,C). In one of the conformers (closed; Fig. 3C,E), the side chain of His356 is on the top of the α-helix and in closer contact with the negatively charged side chains of Glu365 and Glu367, whereas in the second conformer (open; Fig. 3B,D), His356 is closer to another charged side chain, that of Asp353, and the aromatic rings of Phe357 and Tyr331.

The two different orientations of the loops result from the substantial change in the backbone conformation around Phe357, which results in the proximity of Thr358 and Lys354 in the open conformer (Fig. 3B,D). The average backbone torsion angles of Phe357 in the ensemble of the open conformer are −60 ± 3° (*φ*) and −38 ± 4° (*ψ*); these values fall within the range acceptable for an α-helical conformation. In the case of the closed conformer (Fig. 3C,E), these values are +57 ± 3° (*φ*) and +6 ± 4° (*ψ*), which indicates the site of a break in the α-helix (residues 348–356).

**Fig. 4:**
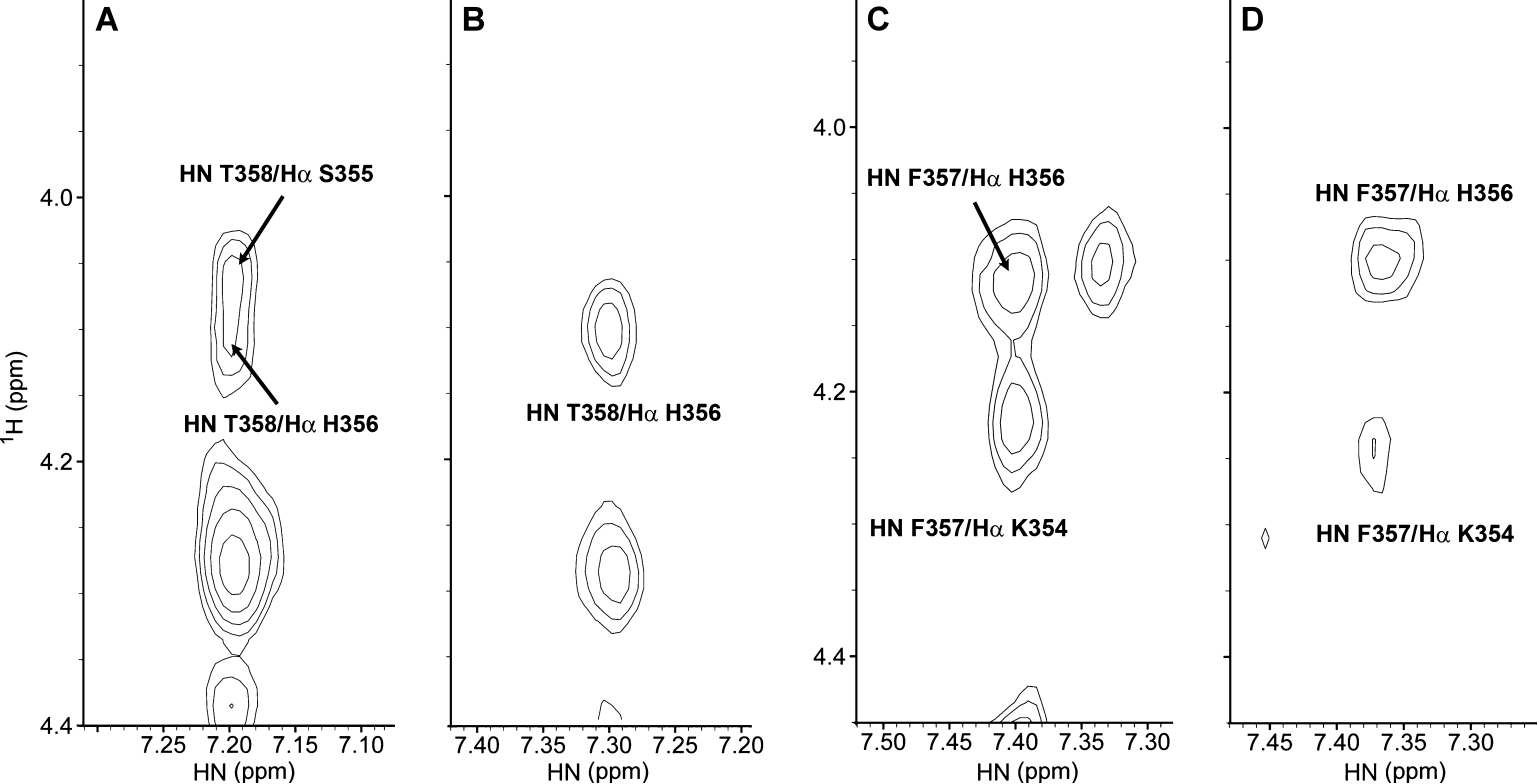
Slices from a ^15^N-HSQC-NOESY spectrum measured at 298 K. The NOEs involving protons of residues from the open conformer (A, C) and closed conformer (B, D) are shown.

The difference between the conformers is clear from the comparison of the intensities of the NOEs involving the HNs of Phe357 and Thr358 (Table S1). Such a twist in the protein backbone conformation between residues 354 and 358 causes a change in the proton–proton distances and the intensities of the corresponding NOEs (Fig. 4). Thus, the NOE between the HN of Thr358 and the Hα of Ser355 could only be detected for the open conformer, whereas a crosspeak between the HN of Thr358 and the Hα of His356 could be seen in both conformers (Fig. 4; Fig. S3). At the same time, the intensity of the NOE between the HN of Phe357 and the Hα of Lys354 in the open conformer is larger than in the closed conformer (Fig. S4). These observations are in full agreement with the structures of the two protein conformations (Fig. 3D,E), calculated with the extensive use of the RDCs for ^1^D_NH_, which greatly helped with the accurate determination of the protein backbone orientation.

The structure of the protein backbone in the central part of loop 357–367 is similar for both conformers, which is in accord with the nearly identical sets of strong long-range, middle-range and intraresidue NOEs found for the two conformers (Fig. S2). There is also no significant change in the conformation of the polypeptide chain in region 365–372. The torsion angle *ψ* of Gln364 differs by 180° in the two protein conformers; however, this does not have a significant impact on the observed interatomic distances, partially owing to the high mobility of this protein region.

### Temperature effects

Raising the temperature from 298 to 313 K leads to a significant decrease in the intensities of all the NOEs arising from the HN of Gly337 and all the sequential and medium-range NOEs arising from the HN atoms of Thr338, Glu339 and Thr358 in the open conformer. However, there is no analogous temperature effect on these signals in the closed conformer. This can be explained by increased mobility of this region in the open conformer, and partially by faster exchange of the amide proton of Gly337 with water. The second suggestion is supported by the existence of strong crosspeaks between the signal of the HN of Gly337 in the open conformer and the signal of the water protons. All of these observations indicate that the loop region of the open conformer has a higher degree of mobility than that in the closed protein conformer.

### Testing the conformer stability

The HSQC spectra of samples of the human C-domain from different preparations showed slightly different relative populations of the two conformers. Therefore, the effects of pH, ionic strength of the solution and temperature on the populations of the two protein conformers were examined (see Experimental procedures). It was found that variation of pH in the range between 6.3 and 7.7, of ionic strength between 25 and 100 mm NaCl and of temperature between 278 and 313 K did not lead to any detectable change in the populations of the two conformers. However, as described earlier, refolding of the protein from a solution at pH 3.5 resulted in only the closed conformer being present in solution. Therefore, one hypothesis is that the protonation state of the His356 side chain could be crucial for protein folding and for the stabilization of the conformers. At pH values above its p*K*_a_, the imidazole ring of His is uncharged, and both conformers are stable. In attempts to experimentally detect any possible pH dependence of the populations of the two conformers, an NMR pH titration of the C-domain in solution was carried out. However, a significant amount of aggregated protein was detected at and below pH 6.0, which precluded the acquisition of this experimental evidence. The fact that protein expression gives equal populations of two protein conformations may also indicate that chaperones and/or cell translation machinery could facilitate the folding of the C-domain.

The relative populations of the two protein conformations were found to be extremely sensitive to the nature of the alignment media used in the RDC experiments (see Experimental procedures). In *n*-alkyl-poly(ethylene glycol)/*n*-alkyl alcohol medium [23], only the closed conformer could be detected. However, in media formed with phospholipid bicelles (DMPC/DHPC and DMPC/DHPC/SDS), the open conformer (90%) was mainly observed.

### Backbone dynamics

**Fig. 5:**
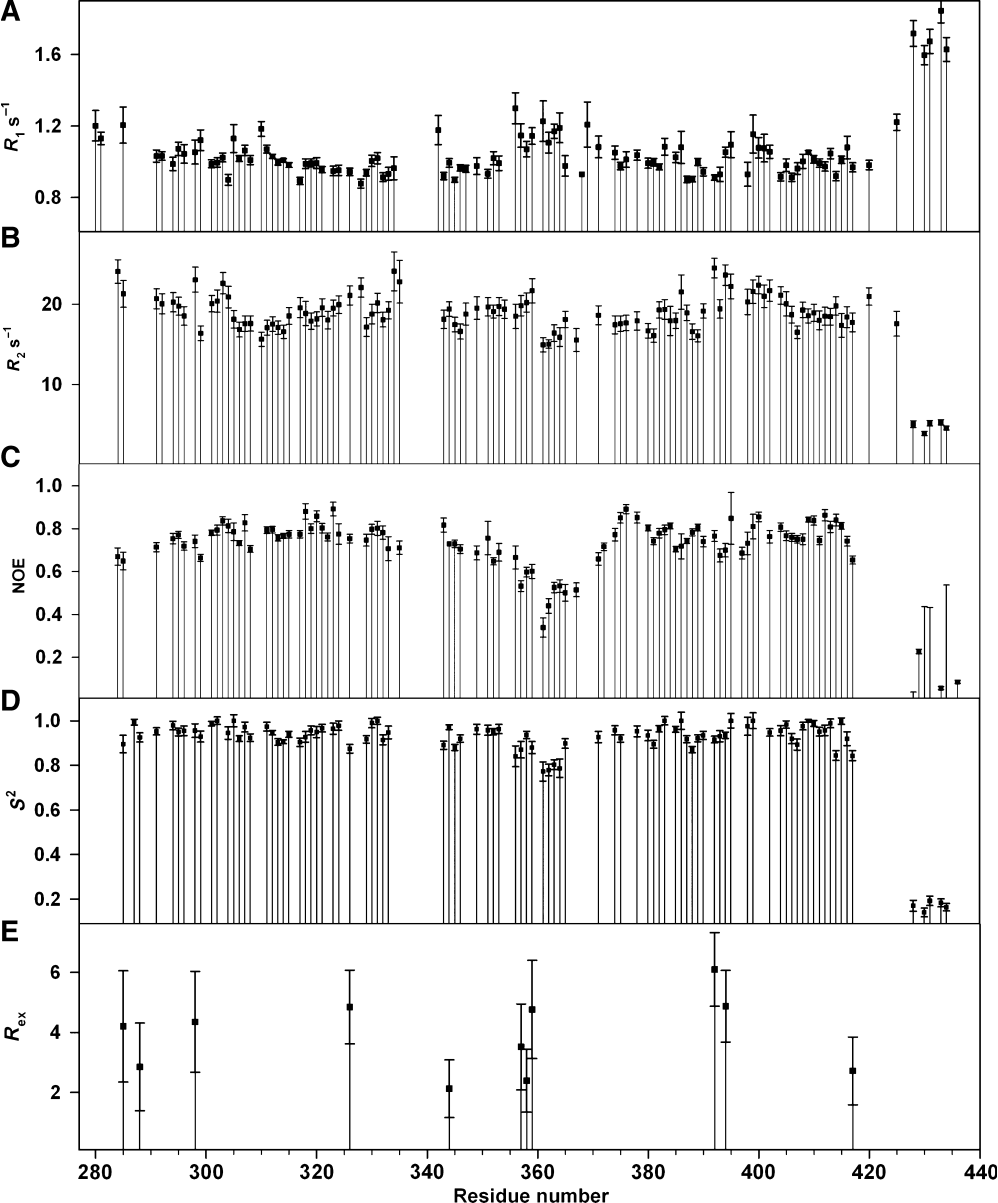
The relaxation parameters of the amide ^15^N nuclei of each residue of the C-domain, measured at 14 T (600 MHz proton resonance frequency) and 298 K. (A) The longitudinal relaxation rate, *R*_1_ (s^−1^). (B) The transverse relaxation rate, *R*_2_ (s^−1^). (C) The heteronuclear ^15^N,^1^H-steady-state NOE values. (D) The order parameter *S*^2^, determined by model-free analysis. (E) Chemical exchange *R*_ex_ contributions to the transverse relaxation rates (s^−1^).

Experimentally determined ^15^N-relaxation parameters for the amide ^15^N nuclei (*R*_1_, longitudinal relaxation rate; *R*_2_, transverse relaxation rate; and ^15^N{^1^H}-NOE values) measured at 298 K are shown in Fig. 5A–C. Figure 5D also shows the calculated values of the order parameter *S*^2^, which reflects the amplitude of picosecond–nanosecond amide bond vector dynamics, and Fig. 5E shows additional line broadening (*R*_ex_) resulting from protein motions on the millisecond time scale. The best fitting of the relaxation parameters could only be obtained using a fully asymmetric tensor model for the molecular rotational diffusion motions.

Analysis of the relaxation data (Fig. 5) shows that, ignoring the trivial case of the C-terminal tail of the protein, the most flexible region in the C-domain is loop 357–367 (Fig. S5). It is important to mention that no noticeable differences in the values of *R*_1_, *R*_2_ and ^15^N{^1^H}-NOE for the two protein conformers, measured at 298 K, were detected. This indicates that the protein backbone mobility on the picosecond–nanosecond time scale is practically identical in the open and the closed conformers of the minidomain (Fig. S6).

A substantial contribution of chemical exchange, *R*_ex_, to the transverse relaxation rate, *R*_2_, was observed for residues 357–359 in the loop region (Fig. 5E). This result is in good agreement with the observed conformational changes between the open and the closed conformers, as Phe357 exhibits the most significant structural perturbation. Structural changes for residues 358 and 359 are smaller, but still detectable.

### Effect of mutations in the minidomain on stop codon specificity

**Fig. 6:**
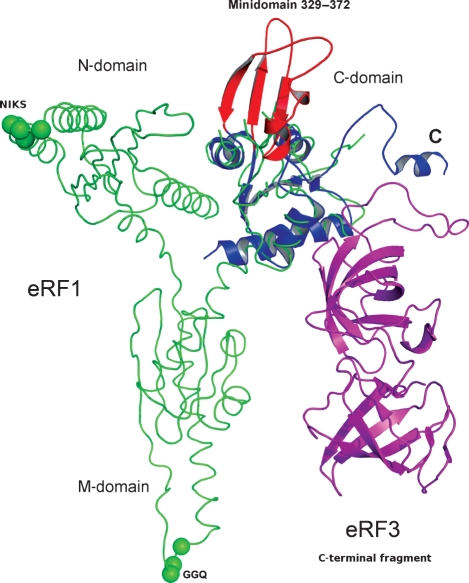
Superposition of the representative NMR open conformer of the C-domain (red and blue) on the crystal structure [20] of the complex of human eRF1 (green) and the truncated eRF3 (purple). The superposition was made using the Cα, C′ and N atoms of the C-domain core residues. The minidomain is shown in red. The top codon recognition NIKS sequence in the N-domain and the strictly conserved GGQ triplet in the M-domain involved in peptidyl-tRNA hydrolysis are indicated by spheres around Cα atoms. The minidomain is close to the N-domain.

**Fig. 7:**
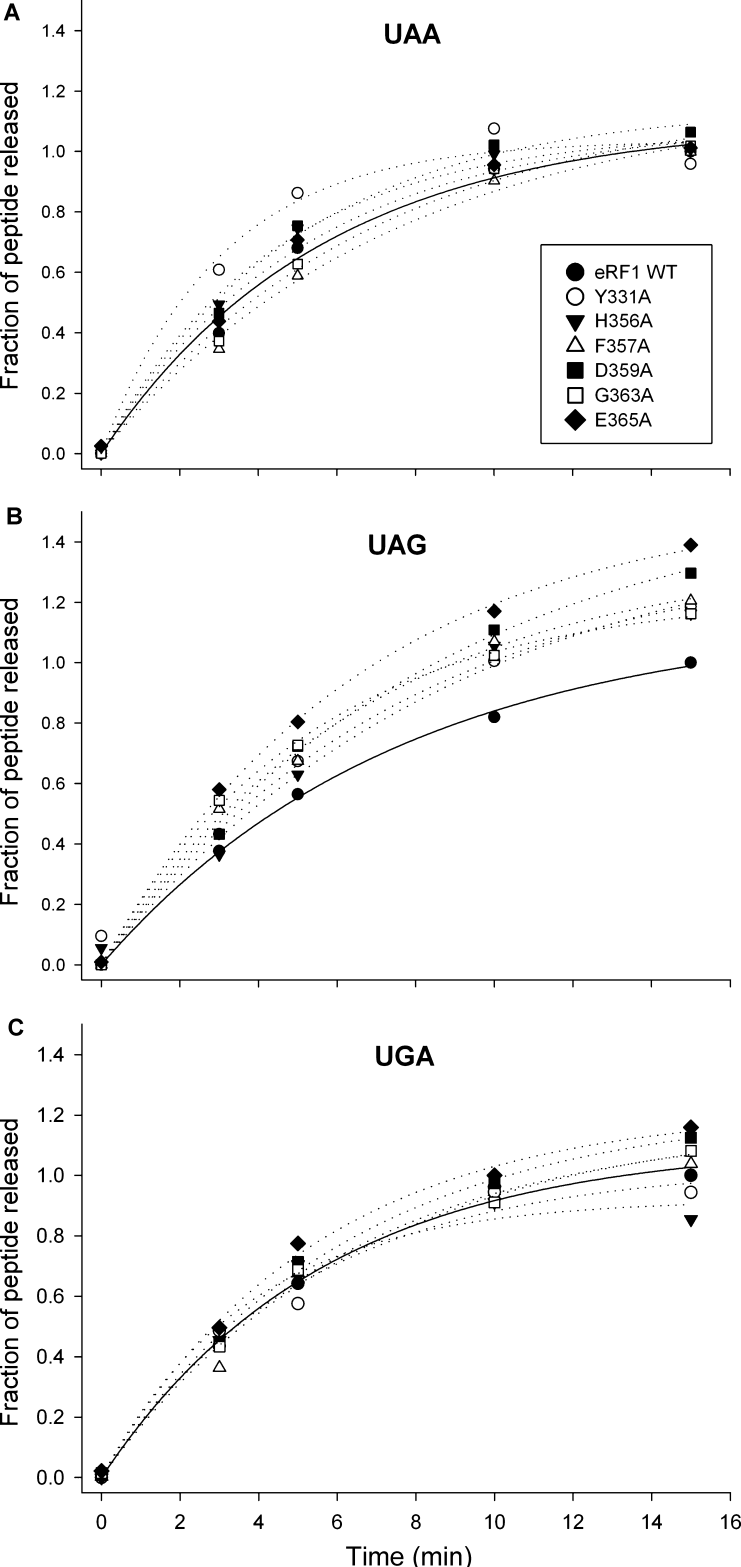
The rate of peptidyl-tRNA hydrolysis in response to human eRF1 with mutations in the minidomain. The ^35^S-labeled tetrapeptide (MVHL) released as a function of time from termination complexes formed with UAA (A), UAG (B) and UGA (C) stop codons by wild-type eRF1 (solid circles) or mutant forms of eRF1 is shown. The background release of tetrapeptide in the absence of eRF1 was subtracted from all graphs. The data are normalized to the release given by wild-type eRF1 at 15 min.

Superposition of the NMR structure of the human C-domain on the full-length crystal structure of eRF1 reveals that the minidomain is located close to or adjacent to the N-domain (see Discussion), which is responsible for the stop codon recognition (Fig. 6). One can assume that complex dynamic behavior of the minidomain may influence the state of the N-domain and may therefore modify the efficiency of the decoding process. To verify this hypothesis, we generated a series of mutant forms of eRF1 with the replacement of Tyr331, His334, His356, Phe357, Asp359, Gly363, Glu365, His366 and Glu370 by alanine. These point mutants were further assayed in a reconstituted *in vitro* eukaryotic translation system containing 60S and 40S ribosomal subunits, mRNA with different stop codons, aminoacylated tRNAs, and individual purified translation factors [13]. The efficiency of termination was estimated from the amount of released ^35^S-labeled peptide at several time intervals. The mutations Y331A, H356A, F357A, D359A, G363A and E365A in the loop region were found to increase the termination efficiency of the ribosomal complex with the UAG stop codon, whereas the peptide release rate did not change significantly when UAA or UGA stop codons were used (Fig. 7). The maximum impact on the peptidyl-tRNA hydrolysis was found for the E365A and D359A mutants, in which negatively charged residues were replaced by alanine. One can speculate that the negative charges reduce the efficiency of the minidomain interaction with mRNA. It is also worth noting that the maximum impact was observed for mutations in the flexible loop 357–367. Replacement of His334, His366 and Glu370 did not change the peptide release rate, regardless of the stop codon used (Fig. S7).

In order to determine whether the observed effects of the mutations could be caused by changes in the efficiency of binding of eRF1 to eRF3, GTPase assays were performed. As eRF3 coupling with eRF1 and the ribosome results in activation of the eRF3 GTPase [4], GTP hydrolysis in such a ternary complex could be used to measure the efficiency of the eRF1–eRF3 interaction. All of the eRF1 mutants stimulated eRF3 GTPase activity nearly identically to that of the wild-type protein (TableS2). These results indicate that the C-domain is able to change the efficiency of stop codon recognition in a context-dependent manner.

## Discussion

### Comparison with crystal structure of human eRF1

The two reported crystal structures of human eRF1 (the protein itself, Protein Data Bank accession code 1DT9; and the complex of eRF1 with eRF3, Protein Data Bank accession code 3E1Y] contain the coordinates of the rigid protein core. However, these structures do not show the coordinates of the atoms in the minidomain, owing to the increased mobility of this protein fragment. The NMR structure of the human C-domain in solution reported here therefore represents the first view of this minidomain. Moreover, it was found that this minidomain exists in two conformations that undergo slow interconversion (on an NMR time scale). The lifetime of these conformational states is certainly longer than seconds, as no noticeable convergence of the two sets of signals was detected, even at 313 K.

**Fig. 8:**
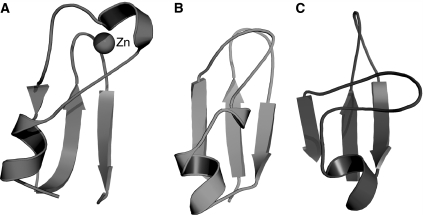
The topology of the zinc-binding domain of zinc finger protein Ynr046w, a component of the yeast eRF1 methyltransferase (A), and the minidomain (residues 329–372) of human eRF1 in the open (B) and closed (C) forms.

Despite the rather simple topology of the minidomain (three antiparallel β-strands and one α-helix on top of the β-sheet), a search of the CATH database (http://www.cathdb.info) [24] provided no direct structural homologs. The closest cluster of structures has the fold found in the factor Xα inhibitor (CATH code 4.10.410). An additional manual search based on these results highlighted a structural homology between the minidomain and the zinc-binding domain of the zinc finger protein Ynr046w [25] (Fig. 8). The fit of the heavy atoms (Cα, C, and N) from three β-strands and the α-helices of both the closed and the open conformer onto a corresponding set of atoms of Ynr046w gives rmsd values of 3.7 and 4.1 Å, respectively. Smaller rmsd values of 1.9 and 2.4 Å are obtained when the β-core residues only are used for the superposition. Interestingly, this protein is a component of yeast eRF1 methyltransferase, which is involved in methylation of the Glu from the strictly conserved GGQ tripeptide, and therefore it also, like human eRF1, plays an important role in translation termination.

The superposition of the families of solution structures of the two conformers onto the crystal structure of human eRF1 (3E1Y) gives an rmsd for the heavy protein backbone atoms (N, Cα, and C′) of 2.81 ± 0.13 Å for all residues of the C-domain except for the highly flexible C-terminal tail (residues 414–437). A superposition made using the same set of atoms from only the residues that belong to the main core of the protein gives a smaller rmsd of 1.58 ± 0.06 Å. Figure 6 shows a comparison of the structure of the protein core (residues 277–328 and 373–413) in solution and in the solid state, and indicates their similarity.

A superposition of the C-domain NMR structure on the crystal structure of the eRF1–eRF3 complex shows that the minidomain is in close proximity to the N-domain (Fig. 6). Recently, a molecular model of the complex of human eRF1 with mRNA and tRNA has been constructed [26]. Among the features of this complex, the authors noted that the C-domain was close to the mRNA stop codon region.

### Stabilization of the two conformers

The two conformational states of the minidomain are almost equally populated, indicating that the energies of formation of these two states should be almost equal. The lifetime of each of the conformational states, and therefore the energy barrier between them, is relatively large. However, gel filtration experiments on the C-domain showed the presence of one peak only (Fig. S8). Therefore, the two protein conformers either have lifetimes of less than a few minutes or have similar physical properties. One can speculate that the two conformational states could be stabilized by the network of coulombic interactions between the charged side chains of the minidomain residues. The minidomain is indeed enriched in polar and charged residues, and the main structural difference between the two conformers is in the relative position of the side chain of His356 with respect to the negatively charged Asp353 and Glu367. The carboxyl groups of these two residues can form hydrogen bonds with the HN proton of the His356 imidazole ring, either directly or through a water molecule. His356 is near Asp353 in the open conformer and near Glu365 and Glu367 in the closed conformer, and these polar interactions may play an important role in the stabilization of the two conformers. The two His residues, His334 and His356, may both participate in stabilization of the polar interactions. Thus, His334, situated on the central β-strand, could interact with the Glu341 and Glu367. A stronger network of interactions between Glu341, His334 (Glu367/Glu365) and His356 in the closed conformer may partially explain why the closed conformer is more rigid than the open one.

The structure of loop 357–367 in both conformers could also be stabilized by hydrogen bonds between the backbone carbonyl oxygen of Asp359 and the amide proton of Gly363 (Fig. S9). The distance between these atoms in the open conformer family is 1.70 ± 0.02 Å, and in the closed conformer it is 1.87 ± 0.10 Å. Additionally, the conformation of this loop could be partially stabilized by the interaction of the carbonyl oxygen of Asp359 with the amide proton of Thr362 (the distance in the open conformer family is 2.34 ± 0.01 Å, and in the closed conformer it is 2.56 ± 0.26 Å) and possibly by the hydrophobic interactions of the methyl groups of Thr358 and Thr362 with a favorably oriented CH_2_ group of Asp359, interactions that were confirmed by the corresponding set of NOEs.

### Dynamic properties of the C-domain

The C-domain reveals a rather complex picture of the mobility of its protein backbone. Analysis of the ^15^N-relaxation data shows that the protein core (residues 277–328 and 373–413) is rather rigid. This is in full agreement with the results of the crystallographic analysis of human eRF1 [19]. The minidomain, which was not resolved in the crystal structures, exists in two conformational states in solution. This is evidence for the existence of protein backbone conformational rearrangements occurring on a time scale of seconds or slower. However, the amplitudes of the motions of the minidomain backbone on the picosecond–nanosecond time scale are rather small, as shown by the large values of the order parameter *S*^2^, which are similar to the corresponding parameters of residues in the protein core region. The most flexible parts of the minidomain are loops 335–339 and 357–367. An accurate analysis of ^15^N-relaxation measurements of residues 335–339 was not possible, owing to the overlapping of peaks in the ^15^N,^1^H-correlation spectra, but the dynamics of loop 357–367 were analyzed. As seen in Fig. 5D, the relative amplitudes of the backbone motions of loop 357–367 were found to be larger than for all the other protein domains except for the C-terminal tail (residues 414–437). Several residues from loop 357–367 also exhibited conformational rearrangements occurring on the millisecond time scale (Fig. 5E). Overall, it seems that the slow conformational triggering is the most characteristic feature of the dynamics of the C-domain.

### Possible functional role of the minidomain

There are several proteins that bind to eRF1. It has been shown by deletion analysis that the catalytic subunit of protein phosphatase 2A (PP2A) binds to region 338–381 of eRF1 [27]. This region substantially overlaps with the minidomain. It is not known whether eRF1 is phosphorylated *in vivo* and whether the interaction of eRF1 with PP2A influences the termination of translation [27]. The eRF1–PP2A interaction may be important for another closely related process, nonsense-mediated decay (NMD). Upf1p, a protein that plays a key role in NMD [28,29], binds to an unknown region of eRF1 [30]. Such an interaction halts translation termination and facilitates the degradation of mRNA [30,31]. It should be noted that the ribonucleoprotein complex, formed during NMD and containing subunits of PP2A, plays a regulatory role in Upf1p phosphorylation.

For termination of translation, eRF3 is one of the most important interaction partners of eRF1. It has been shown previously that both the M-domain [21] and the C-domain [10,11,32] interact with eRF3. Recently, it was also shown that the eRF1 interaction interface with eRF3 is formed by two Arg residues, Arg192 and Arg203, in the M-domain [21] and by a cluster of hydrophobic residues, Phe291, Ile294, Tyr301, Phe303, and Phe406, in the C-domain [20]. Residues 329–372 are situated on the opposite side of the C-domain, and therefore do not participate directly in the interaction with eRF3. This conclusion was also confirmed by the results of the GTPase assays, which showed that mutations in the minidomain of eRF1 did not change the GTPase activity of eRF3.

The minidomain in the crystal structure is near the N-terminal domain, which plays a key role in stop codon recognition. The ability of the minidomain to act as a conformational switch and its probable proximity to the stop codon recognition site in the termination complex hint at its possible functional role. The termination efficiencies of several eRF1 mutants were examined. The residues for mutation were selected from those that appeared to be important for stabilization of the two protein conformers, i.e. those in loop 357–367 and several neighboring residues. The observed impact of the mutations at Tyr331, His356, Phe357, Asp359, Gly363 and Glu365 on the termination efficiency of eRF1 with regard to UAG stop codon recognition are in accord with the hypothesis that the C-domain could be involved in the regulation of translation termination. As Asp359 and Gly363 are important for stabilization of both conformations of loop 357–367, it is possible that stop codon specificity is regulated by the conformation of this flexible part of the C-domain. His334, His366 and Glu370 are located outside this loop, and this may explain the absence of an effect of their replacement by Ala on termination efficiency. Although the effects of the mutations on peptide release are relatively modest, the increase in efficiency (rather than a decrease) is nevertheless an important observation, and makes it more likely that the phenomenon is caused by a direct interaction related to the UAG stop codon recognition process.

It has also been reported that mutations in eRF3 that reduce its GTPase activity also decrease the efficiency of translation termination for some, but not other, stop codons [14]. Thus, a 17-fold reduction in termination efficiency was observed for the UGAC stop signal, whereas much weaker effects were detected in the case of other termination signals. The authors suggested that the GTPase activity of eRF3 acts to couple the recognition of translation termination signals by eRF1 to efficient polypeptide chain release. Genetic screening experiments also identified mutants with changes in the C-terminal tail of yeast eRF1 that were unable to recognize one of the three stop codons [5]. Two of the mutations (Q415X and E428Q) are situated near the eRF3-binding motif, and could therefore influence the efficiency of the eRF1–eRF3 functional interaction [9,12]. The mutations in the minidomain reported here have no impact on the eRF1–eRF3 interaction, and are more likely to control termination efficiency through a direct interaction with the stop codon recognition sites.

It should be noted that loop 357–367 is one of the most variable regions in the sequence of class 1 eukaryotic release factors [33] (Fig. S10). The majority of eukaryotes utilize all three stop codons. However, the frequencies of UAA, UAG and UGA in the coding sequences of mRNAs differ between species [34]. It is possible that the variable residue composition of loop 357–367 may contribute to the modulation of the affinity of eRF1 for different stop codons according to the most abundant termination signal in the transcriptome. Indeed, UAG is a rare stop codon in the *Homo sapiens* transcriptome [34], and is therefore a relatively weak signal for human eRF1. Mutations in loop 357–367 that increase the efficiency of eRF1 recognition of the UAG codon are in agreement with this hypothesis.

## Experimental procedures

### Sample preparation

The DNA fragment encoding the C-domain (residues 277–437) with a C-terminal His_6_-tag fusion was cloned into the pET23b(+) vector (Novagen) under the phage T7 RNA polymerase promoter. The C-domain was overproduced in *Escherichia coli* BL21(DE3), in M9 minimal medium, and isolated using Ni^2+^–nitrilotriacetic acid resin (Qiagen). The protein was further purified by cation exchange chromatography, using HiTrap SP columns (GE Healthcare). For ^13^C and/or ^15^N labeling, [^13^C_6_]d-glucose and/or ^15^NH_4_Cl (Cambridge Isotope Laboratories) were used as the isotope sources in M9 minimal medium. The samples for NMR (protein concentration of ∼ 1 mm) were prepared in either 95% H_2_O/5% D_2_O or 100% D_2_O and 10 mm potassium phosphate and 50 mm KCl (pH 7.0). β-Mercaptoethanol (∼ 2 mm) was added to the final solution in order to prevent oxidation of the free Cys residues Cys302 and Cys335. Shigemi microcell NMR tubes, containing 330–380 μL, were used in the recording of the NMR spectra.

### Cloning and mutagenesis of human eRF1

Plasmids with mutant eRF1 genes were obtained by site-directed mutagenesis, using the PCR-based ‘megaprimer’ method as described previously [6]. The resulting PCR products were inserted into the *Xho*I–*Bst*98I sites of the pERF4b plasmid. The sequences of the PCR primers used for the generation of the eRF1 mutants are available upon request.

### Expression and purification of human RFs

Wild-type human eRF1, its mutants and eRF3c containing His_6_-tags at the C-termini were produced in *E. coli* BL21(DE3), and purified as described previously [6,13,35].

### Purification of initiation and elongation factors, ribosomal subunits, and aminoacylation of tRNA

These are described elsewhere [12,36–39].

### mRNA transcripts

mRNA was transcribed by T7 RNA polymerase on MVHL-stop plasmids, encoding a T7 promoter, four CAA repeats, the β-globin 5′-UTR, the MVHL tetrapeptide followed by one of three stop codons (UAA, UAG, or UGA) and the 3′-UTR, comprising the rest of the natural β-globin coding sequence. The MVHL-stop plasmids (containing UAA, UAG and UGA stop codons) were prepared as described previously [39]. For run-off transcription, all plasmids were linearized with *Xho*I.

### Pretermination complex assembly and purification

Pretermination complexes were assembled as described previously [12,39]. Briefly, 37 pmol of MVHL-stop mRNA was incubated in buffer A (20 mm Tris/acetate, pH 7.5, 100 mm potassium acetate, 2 mm dithiothreitol), supplemented with 400 u of RNase inhibitor (RiboLock, Fermentas), 1 mm ATP, 0.25 mm spermidine, 0.2 mm GTP, 75 μg of total tRNA (acylated with Val, Hist, Leu, and [^35^S]Met), 75 pmol of 40S and 60S purified ribosomal subunits, 125 pmol each of eIF2, eIF3, eIF4F, eIF4A, eIF4B, eIF1, eIF1A, eIF5, and eIF5B, 200 pmol of eEF1H and 50 pmol of eEF2 for 30 min, and then centrifuged in a Beckman SW55 rotor for 95 min at 4 °C and 300 000 ***g*** (using a Beckman SW55 rotor) on a 10–30% linear sucrose density gradient prepared in buffer A with 5 mm MgCl_2_. Fractions corresponding to pretermination complexes, according to their optical density and the presence of [^35^S]Met, were combined, diluted three-fold with buffer A containing 1.25 mm MgCl_2_ (to a final concentration of 2.5 mm Mg^2+^), and used for the peptide release assay.

### Peptide release assays

These were performed as described previously [12], with some minor modifications. Aliquots containing 0.1 pmol of pretermination complexes, formed in the presence of [^35^S]Met-tRNA, and with an activity of about 10 000 c.p.m., were incubated at 37 °C with 2.5 pmol of eRF1 for 0–15 min. Ribosomes and tRNA were pelleted with ice-cold 5% trichloroacetic acid, supplemented with 0.75% casamino acids, and centrifuged at 4 °C and 14 000 ***g***. The amount of released [^35^S]Met-containing tetrapeptide, which indicated the efficiency of peptidyl-tRNA hydrolysis, was determined by scintillation counting of the supernatants using an Intertechnique SL-30 liquid scintillation spectrometer.

### GTPase activity assays

These were based on the measurement of the accumulation of [^32^P]P_i_, using a modified charcoal precipitation method [7]. The incubation mixture (12.5 μL) contained 20 mm Tris/HCl (pH 7.5), 30 mm NH_4_Cl, 15 mm MgCl_2_, 0.16 μm ribosomes, 0.16 μm human eRF3c, and 0.5 μm [^32^P]GTP[γP] (10 000 c.p.m./pmol); human wild-type eRF1 or mutant eRF1s were added to give 0.04, 0.08, 0.12 and 0.16 μm final concentrations. The reactions were run at 30 °C for 20 min, and terminated by mixing with 0.5 mL of a 5% activated charcoal suspension in 50 mm NaH_2_PO_4_, cooled on ice. The mixture was vortexed and centrifuged at 16 000 ***g*** for 10 min at 4 °C. Aliquots of the supernatants (0.375 mL) were counted on a scintillation counter. Values of eRF3 GTPase activity and corresponding error limits were estimated from five experiments carried out for each eRF1 mutant.

### Gel filtration analysis of the C-domain

This was performed on Superose 12 in a buffer containing 20 mm Tris/HCl (pH 7.5), 100 mm KCl, 2 mm dithiothreitol, and 5% glycerol. Only one peak was observed, indicating that the two conformational states could not be separated by this method.

### NMR spectroscopy

All spectra were acquired on Varian INOVA 600 and 800 MHz and Bruker AVANCE 600 and 700 MHz spectrometers equipped with triple-resonance *z*-gradient probes. The 700 and 800 MHz spectrometers were equipped with cryoprobes. Spectra were processed by nmrpipe, and analyzed using sparky (from Goddard and Kneller; http://www.cgl.ucsf.edu/home/sparky) and autoassign [40]. Sequential backbone assignments [41] and side chains assignments were obtained using 3D spectra obtained from 3D experiments correlating the amide NH with the C′ signal of the preceding amino acid, correlating the amide HN and the Cα signals, correlating the amide HN and the Cα signal of the preceding amino acid, correlating the amide NH with the Cα and Cβ signals, correlating the amide NH with the Cα and Cβ signals of the preceding amino acid, a three-dimensional experiment correlating amide HN with Ha and Hb signals (HNHAHB), three-dimensional experiment correlating amide HN with Ha and Hb signals of preceding residue via carbonyl carbon (HBHA(CO)NH) and three-dimensional experiment correlating amide HN and Ha signals (HNHA) [42], measured at 298 K, and three-dimensional experiment correlating side-chain protons via ^13^C-^13^C correlations (HCCH)-TOCSY, measured at 313 K. Additional side chain assignments and NOE distance restraints were extracted from the ^1^H,^13^C-NOESY and ^1^H,^15^N-NOESY spectra measured at 298 and 313 K with 100 ms mixing time. Assignments were obtained for more than 99% of the ^1^H, ^13^C and ^15^N atoms of the protein backbone, and for more than 78% of the side chain atoms.

The main set of backbone *ϕ* and *ψ* dihedral angles was calculated from the chemical shift values of backbone atoms ^13^Cα, ^13^Cβ, ^13^C′, ^1^Hα, ^1^HN, and ^15^N, using talos software [43]. Additional dihedral angles for those residues with no agreement in talos were obtained by the anglesearch program [44].

RDC constants were measured using partially oriented diluted liquid crystalline media: ∼ 5% (v/w) C12E5/hexanol [23] and DHPC/DMPC bicelles [22]. In this series of experiments, alternative orientations of the alignment tensor were achieved by modifying the DHPC/DMPC bicelles with SDS. Sixty-nine RDCs were measured in C12E5/hexanol, and 90 in DHPC/DMPC/SDS, at 311 K. Neutral 5% (v/w) DHPC/DMPC bicelles were also used. However, none of these measured RDCs was used in the subsequent calculations, because of the very weak alignment of the protein in this medium (maximum dipolar interactions did not exceed 5 Hz). The RDC values were calculated from the ^1^DJ_NH_ and ^1^J_NH_ constants, extracted from the inphase antiphase (IPAP)-HSQC spectra [45], acquired in anisotropic and isotropic conditions respectively.

Spectra for the measurement of ^15^N longitudinal relaxation rates (*R*_1_), transverse relaxation rates (*R*_2_) and ^15^N{^1^H} heteronuclear NOE values were collected on a ∼ 1 mm^15^N-labeled C-domain sample at 298 K with a Varian INOVA 600 MHz NMR spectrometer, using pulse sequences modified from those described by Kay *et al.* [46] to compensate for cross-correlation effects [47].

### Structure calculation and refinement

The initial structure was generated in cns, using a set of manually unambiguously assigned NOEs. The structure was then submitted to aria, and further assigned NOEs were obtained by an iterative procedure [48] using aria-cns [49]. NOE peak intensities were used for distance estimation, instead of volumes, because of significant crosspeak overlapping. All of the measured proton–proton distances were divided into ranges, with upper limits of 2.5, 3.0, 3.5, 4.0, 4.5, 5.0, 5.5 and 6.0 Å. The structure calculations and refinement were performed by a simulated annealing protocol carried out in Cartesian coordinate space using cns [50] and the slightly modified script anneal.inp. The calculations were performed in an iterative manner. Database values of conformational torsion angle pseudopotentials [51] were introduced at the final stages of refinement. The final force constants were as follows: NOE restraints, 75 kcal·mol^−1^·Å^2^; dihedral angle restraints, 200 kcal·mol^−1^·rad^2^; RDCs, 50 kcal·mol^−1^·Hz^2^; and a scale factor for conformational database restraints [10,51]. The weighting for the RDC potential was scaled from 0.01 to 50. The restraint violations were monitored after each cycle of refinement by the in-house program nmrest or the cns script accept.inp. Violated restraints were checked and corrected or declined. One thousand eight hundred and fifty-seven NOE-derived distance restraints, 216 dihedral angles and 90 RDCs were used in the calculation of the final ensemble (Table 1). The structure quality was analyzed with aqua and procheck-nmr software [52] (Fig. S11) and by using the nmrest program. The best 24 structures out of 100 (with respect to the minimum restraints violation value criterion) were accepted as the final ensemble for each protein conformer.

Structure visualization and analysis were carried out using the insightii software package (Accelrys Software Inc.) and pymol (DeLano Scientific LLC).

### NMR dynamics analysis

*R*_1_, *R*_2_ and ^1^H,^15^N-heteronuclear NOE datasets of the C-domain uniformly labeled with ^15^N were collected at 298 K on a 600 MHz Varian Inova spectrometer. The delays for the *R*_1_ experiments were 0.6, 8.6, 24.7, 48.8, 96.9, 193.2, 345.7, 498.2, 795.2, 1196.4 and 1597.3 ms, and those for the *R*_2_ experiments were 0, 8.6, 17.2, 25.8, 34.4, 43.0, 51.7, 60.3, 77.5 and 94.7 ms. The excitation time for ^1^H in the ^1^H,^15^N-heteronuclear NOE experiments was 4.0 s. Spectra were processed using nmrpipe [53]. The nonlinear fitting of the integrated peak volumes in the pseudo-3D spectra of the relaxation rate experiments and the calculation of standard deviations were accomplished using the nlinls procedure. The values of *R*_1_ and *R*_2_ were then calculated from the table of relative peak intensities, produced by nmrpipe and nlinls, using relaxfit, which was written in-house [54]. The standard deviations of the ^15^N{^1^H}-NOE values were calculated using the rmsd noise of the background regions [55], and were further checked and corrected by using two independently collected experimental datasets.

The analysis of the *R*_1_, *R*_2_ and ^1^H,^15^N-NOE values was carried out using a model-free formalism, with tensor 2.0 [56]. To determine the rotational diffusion tensor, all of the isotropic, axially symmetric and fully asymmetric molecular tumbling models were tested. Parameters of the tensors for fully anisotropic diffusion of the open and closed conformers are presented in Table 2. The values of the diffusion tensor axis were then used to fit models of internal motions for the backbone HN vectors of the amino acids. Five models were tested during the calculation: (a) a rigid body model (using the very fast internal motions, *t*_c_ < 20 ps) (model 1); (b) the model-free Lipari–Szabo model [57] (model 2); (c) the Lipari–Szabo model with the inclusion of the chemical exchange contribution, *R*_ex_, to the transverse relaxation rates [58] (model 3); (d) a rigid body model with the inclusion of the chemical exchange contribution (model 4); and (e) the model-free Lipari–Szabo model with an extension to include slower internal motions occurring on a nanosecond time scale [59] (model 5).

**Table 2:** Parameters of the diffusion tensors for anisotropic tumbling of the open and closed conformers of the C-domain at 298 K, calculated by tensor 2.0 [56].

Tensor parameter	Open conformer	Closed conformer
*D*_*xx*_ = 10^8^ (s^−1^)	0.102 ± 0.002	0.101 ± 0.002
*D*_*yy*_ = 10^8^ (s^−1^)	0.125 ± 0.003	0.128 ± 0.002
*D*_*zz*_ = 10^8^ (s^−1^)	0.140 ± 0.003	0.139 ± 0.002
*τ*_1_ = (4*D*_*xx*_ + *D*_*yy*_ + *D*_*zz*_)^−1^ (ns)	14.86 ± 0.18	14.90 ± 0.27
*τ*_2_ = (*D*_*xx*_ + 4*D*_*yy*_ + *D*_*zz*_)^−1^ (ns)	13.48 ± 0.22	13.30 ± 0.21
*τ*_3_ = (*D*_*xx*_ + *D*_*yy*_ + 4*D*_*zz*_)^−1^ (ns)	12.71 ± 0.19	12.74 ± 0.19

Typically, most of the residues of the protein rigid core could be successfully fitted using models 1 and 2. For a few residues of the minidomain (330–332, at the beginning of the central β-strand) and residues 343 and 351, model 1 was selected as the best. For residues 357–359 of the minidomain and for several other residues, a significant contribution of chemical exchange was observed, and model 3 had to be used to fit the relaxation data. For the other residues of the minidomain, model 2 was selected. Model 5 was applied only to fit the relaxation data obtained for the residues from the flexible C-terminal tail.

## Abbreviations

C-domain: C-terminal domain (or domain 3) of class 1 polypeptide chain release factor
DHPC: 1,2-dihexanoyl-*sn*-glycero-3-phosphocholine
DMPC: 1,2-dimyristoyl-*sn*-glycero-3-phosphocholine
eRF1: eukaryotic class 1 polypeptide chain release factor
eRF3: eukaryotic class 2 polypeptide chain release factor 3
HSQC: heteronuclear single quantum coherence
M-domain: eukaryotic class 1 polypeptide chain release factor middle domain (or domain 2)
minidomain: residues 329–372 of human eRF1
N-domain: eukaryotic class 1 polypeptide chain release factor N-terminal domain (or domain 1)
NMD: nonsense-mediated decay
PP2A: protein phosphatase 2A
RDC: residual dipolar coupling
*R*_ex_: conformational exchange contribution to *R*_2_
RF: release factor
*R*_1_: longitudinal or spin–lattice relaxation rate
*R*_2_: transverse or spin–spin relaxation rate
*S*^2^: order parameter reflecting the amplitude of picosecond–nanosecond bond vector dynamics
*τ*_e_: effective internal correlation time
*τ*_m_: overall rotational correlation time

## Supporting information

The following supplementary material is available:

**Fig. S1.** A region of the ^1^H,^15^N-HSQC spectrum of the C-terminal domain of human eRF1 illustrating the presence of two conformational states of the protein.

**Fig. S2.** Plot of the number and distribution of the NOEs versus the amino acid sequence that were used in the structure calculation for the open and closed conformers of the C-terminal domain of human eRF1.

**Fig. S3.** NOE map of the minidomain (residues 329–372) of human eRF1.

**Fig. S4.** Representative NOEs in the open and closed conformers of the minidomain of human eRF1.

**Fig. S5.** A cylindrical ribbon representation of the backbone of the C-terminal domain of human eRF1.

**Fig. S6.** The order parameter, *S*^2^, calculated separately for the open and closed conformers of the C-terminal domain of human eRF1 using a model-free analysis with an assumption of fully asymmetric molecular motions and tensors.

**Fig. S7.** The rate of peptidyl-tRNA hydrolysis in response to human eRF1, with mutations in the minidomain (H334A, H366A, and E370A).

**Fig. S8.** Results of the gel filtration and SDS/PAGE (8% acrylamide) of the C-terminal domain of human eRF1.

**Fig. S9.** The stabilization of the loop (residues 358–363) by a network of hydrogen bonds.

**Fig. S10.** Multiple sequence alignment of eRF1.

**Fig. S11.** The Ramachandran map plot (*φ* and *ψ* torsion angles for the protein backbone) of all 24 conformers of the NMR families of solution structures of the closed and open conformers of the C-terminal domain of human eRF1.

**Table S1.** Differences in the experimental restraints used for the structural determination of the two conformers of the C-terminal domain of human eRF1 in solution.

**Table S2.** Impact of mutations of human eRF1 on the GTPase activity of eRF3 in its ternary complex with the ribosome.

This supplementary material can be found in the online version of this article.

Please note: As a service to our authors and readers, this journal provides supporting information supplied by the authors. Such materials are peer-reviewed and may be re-organized for online delivery, but are not copy-edited or typeset. Technical support issues arising from supporting information (other than missing files) should be addressed to the authors.
