# Supplementary material for: NMR solution structure and function of the C-terminal domain of eukaryotic class 1 polypeptide chain release factor
Source: FEBS J. 2010 Jun;277(12):2611–27. doi: 10.1111/j.1742-4658.2010.07672.x (PMC2909394; doi:10.1111/j.1742-4658.2010.07672.x)

the FEBS Journal
www.febsjournal.org

NMR solution structure and function of the C-terminal domain of eukaryotic class 1 polypeptide chain release factor

Alexey B. Mantsyzov, Elena V. Ivanova, Berry Birdsall, Elena Z. Alkalaeva, Polina N. Kryuchkova, GeoffKelly, Ludmila Y. Frolova and Vladimir I. Polshakov

DOI: 10.1111/j.1742-4658.2010.07672.x

Supplementary Material

NMR Solution Structure and Function of the C-terminal Domain of Eukaryotic Polypeptide Release Factor eRF1

Alexey B. Mantsyzov, Elena V. Ivanova, Berry Birdsall, Elena Z. Alkalaeva, Polina N. Kryuchkova, Geoff Kelly, Ludmila Yu. Frolova and Vladimir I. Polshakov

Table S1 Impact of mutations of human eRF1 on the GTPase activity of eRF3 in its ternary complex with the ribosome. Activity of the wild type eRF1 is accepted as 100%.

	**GTPase activity, %**
**eRF1 WT**	100
**Y331A**	98 ± 2
**H334A**	98 ± 2
**H356A**	95 ± 4
**F357A**	100 ± 5
**D359A**	97 ± 8
**G363A**	98 ± 6
**E365E**	96 ± 7
**H366A**	97 ± 4
**E370A**	95 ± 6

Table S2 Differences in the experimental restraints used for the structural determination of the two conformers of the C-terminal domain of human eRF1 in solution.

	”Open” conformer	”Closed” conformer
Total number of NOEs	1857	1852
Number of long-range NOEs	497	490
Number of RDCs	90	69
NOEs different between the two conformers<br>(NOE constrained interatomic distance shown in brackets, Å)	$H\alpha^{354}$ – HN^{357} (4.0)<br>$H\alpha^{356}$ – HN^{358} (5.0)<br>$H\alpha^{355}$ – HN^{358} (4.0)<br>HN^{359} – HN^{364} (3.0)<br>HN^{334} – $H\alpha^{368}$ (3.5)<br>$H\alpha^{357}$ – $H\delta_2^{366}$ (4.5)	$H\alpha^{354}$ – HN^{357} (4.5)<br>$H\alpha^{356}$ – HN^{358} (4.0)<br>$H\alpha^{355}$ – HN^{358} (not observed)<br>HN^{359} – HN^{364} (not observed)<br>HN^{334} – $H\alpha^{368}$ (not observed)<br>$H\alpha^{357}$ – $H\delta_2^{366}$ (not observed)

Fig. S1 A region of the $^{1}H,^{15}N$-HSQC spectrum of the C domain of human eRF1 illustrating the presence of two conformational states of the protein. The amide signals from residues which belong to the open conformation are marked with asterisks.

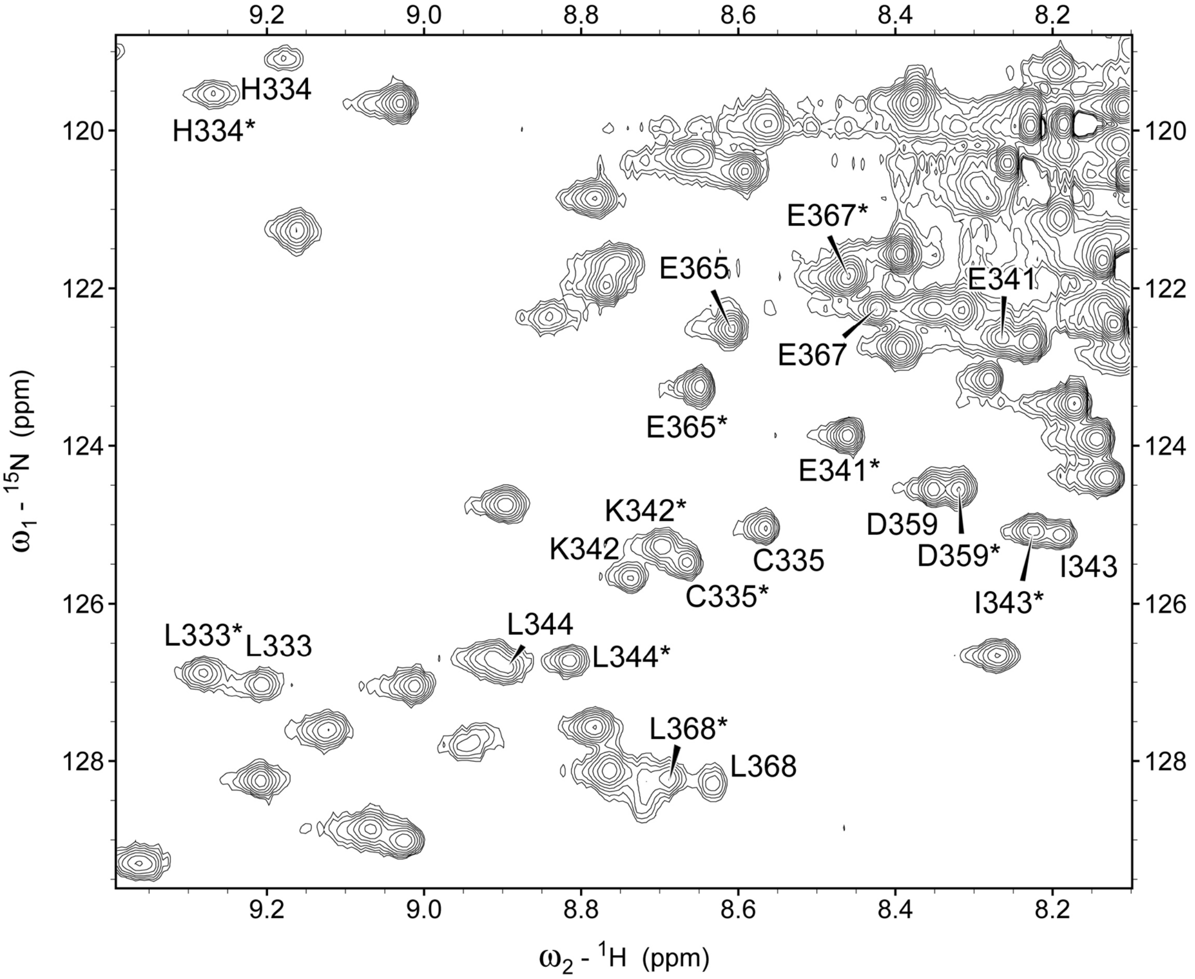

Fig. S2 Plot of the number and distribution of the NOEs verus the amino acid sequence that were used in the structure calculation of the open (A) and closed (B) conformers of the C domain of human eRF1. Each NOE is counted twice (for each residue in the NOE). NOEs are classified as: intra residue (black); sequential (blue, (i-j) = 1); medium range (red, (1 < i-j 4); long range (green, (i-j > 4)).

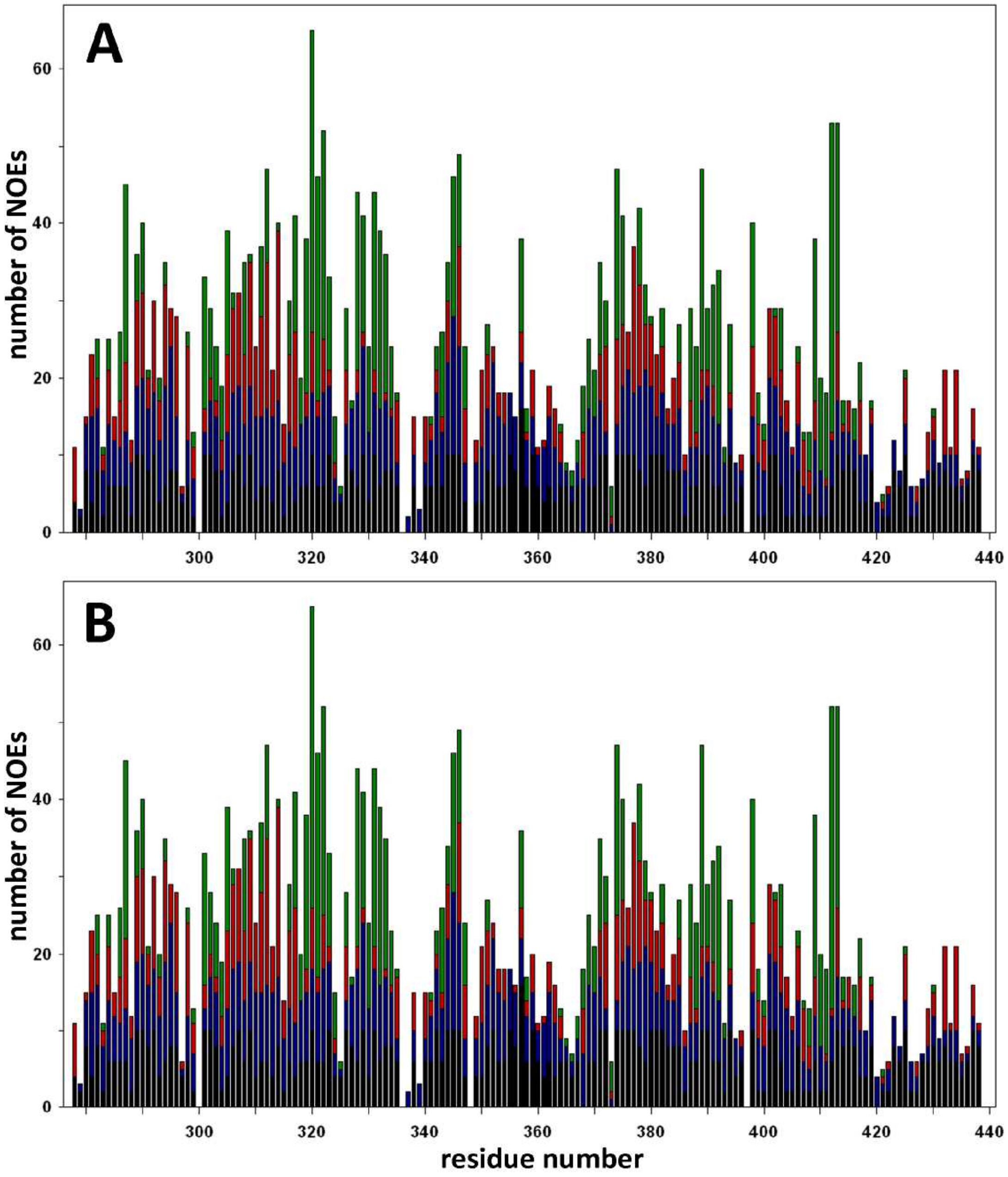

Fig. S3 NOE map of the mini-domain (residues 329-372) of human eRF1. The dots below the diagonal correspond to the NOEs between the protein backbone atoms (Ha and HN). The dots above the diagonal correspond to the observed NOEs between all protons. NOEs observed only in the open conformer are shown in green. NOEs different in intensity between the open and closed conformers are shown in red. The figure was generated using the program NMRest, written in-house.

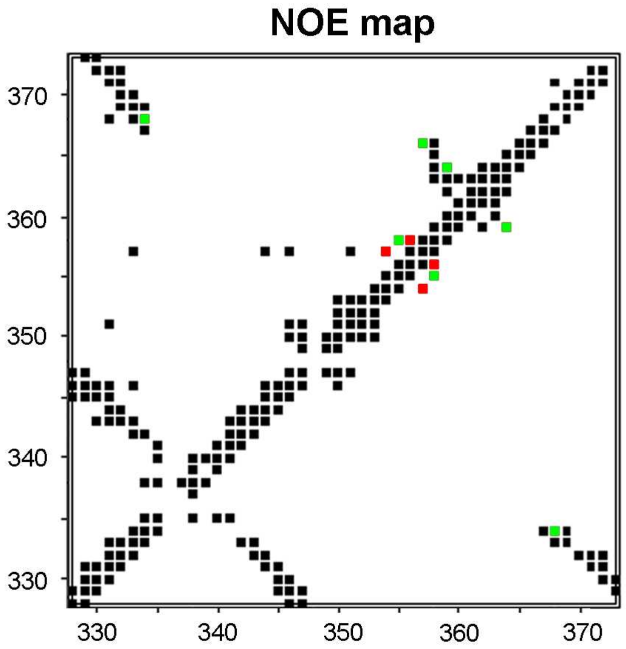

Fig. S4 Representative NOEs in the open (A) and closed (B) conformers of the mini-domain of human eRF1. In the open conformer (A) the distance between K354 Hα and F257NH is 3.3 Å whereas in the closed conformer (B), this distance is larger, 3.5 Å. as manifest in its smaller observed NOE. The distance between T358 NH and H356 Hα and Hβ is also different in conformers A and B.

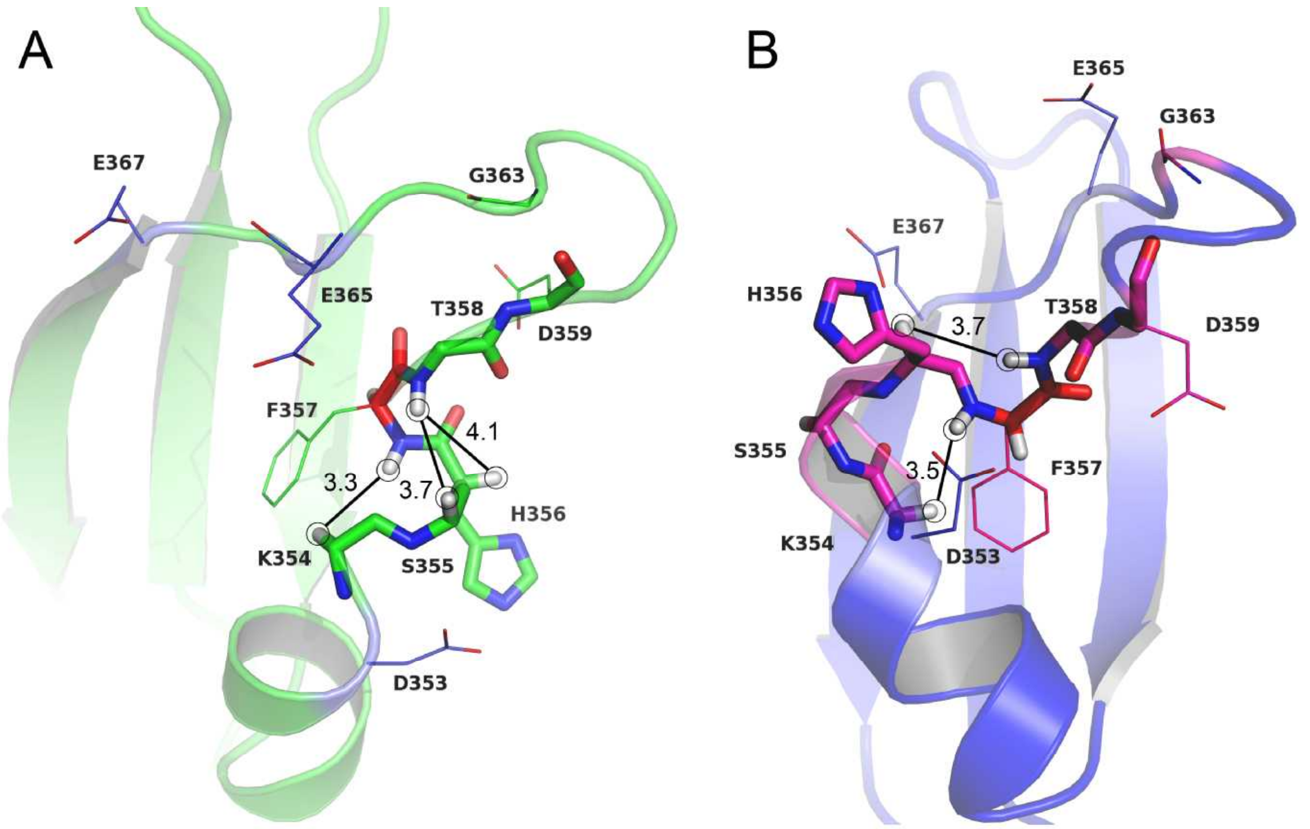

Fig. S5 A cylindrical ribbon representation of the backbone of the C domain of human eRF1. The variable radius/thickness of the cylinder is proportional to the dynamic properties (on the ps to ns time scale) of the protein residues and is proportional to the value of $(1-S^2)$; the minimal thickness corresponds to the value $S^2 = 1$, the maximum to $S^2 = 0.7$. The C-terminal tail (residues 414-437) is not shown.

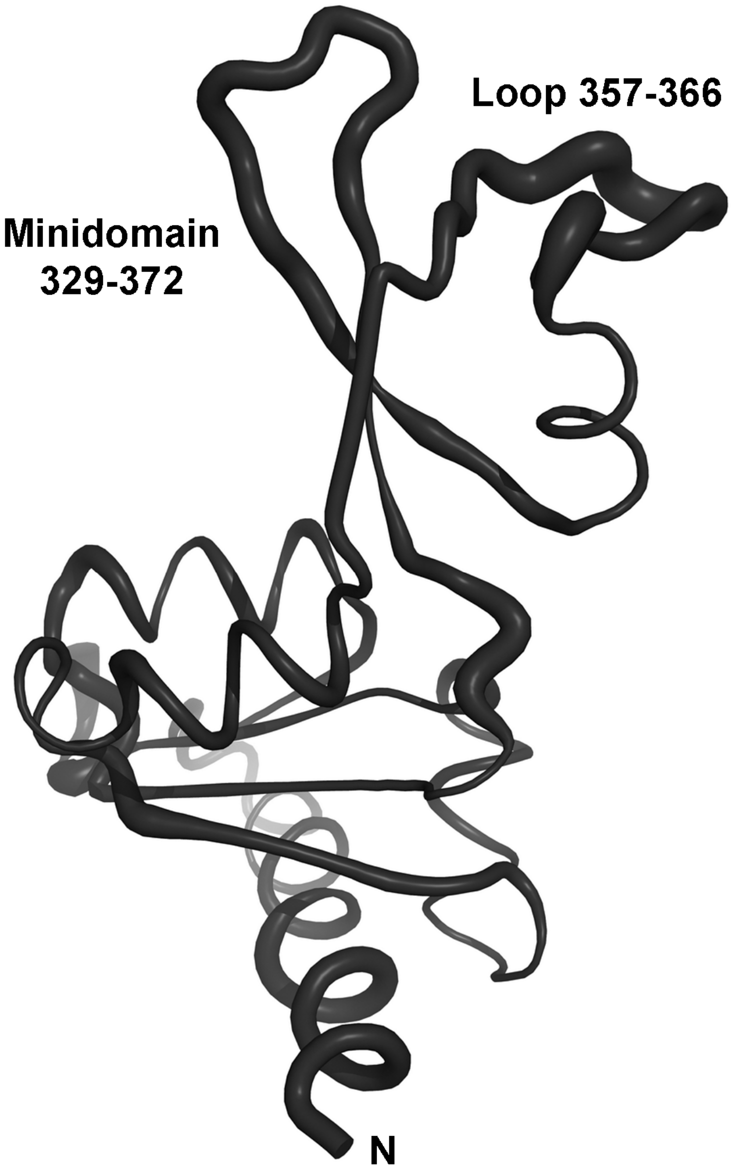

Fig. S6 The order parameter, S^2, calculated separately for the open and closed conformers of the C-domain of human eRF1 using a model-free analysis with an assumption of fully asymmetric molecular motions and tensors. Blue dots represent the values for the open conformer and red dots for the closed conformer. Clearly the order parameters are very similar and the protein motions along the chain are similar for the open and closed conformers.

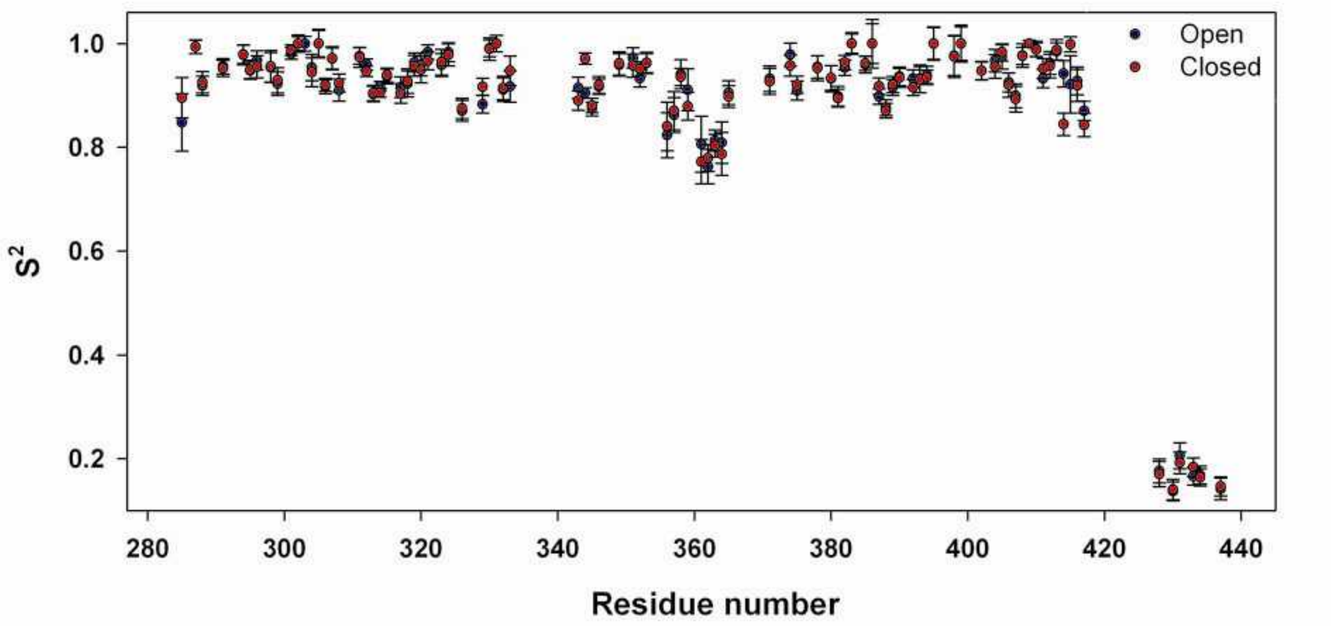

Fig. S7 The rate of peptidyl-tRNA hydrolysis in response to human eRF1, with mutations in the mini-domain 329-372. The fraction of the ^{35}S-labelled tetrapeptide (MVHL) released as a function of time from termination complexes formed with UAA (A), UAG (B), UGA (C) stop codons by the wild type eRF1 (solid circles) or mutant forms of eRF1: His334→Ala (open circles), His366→Ala (solid triangles) and Glu370→Ala (open triangles) is shown. The background release of tetrapeptide in the absence of eRF1 has been subtracted in all graphs. A value of the fraction of peptide released equal to 1 corresponds to the maximum value found for wild type eRF1.

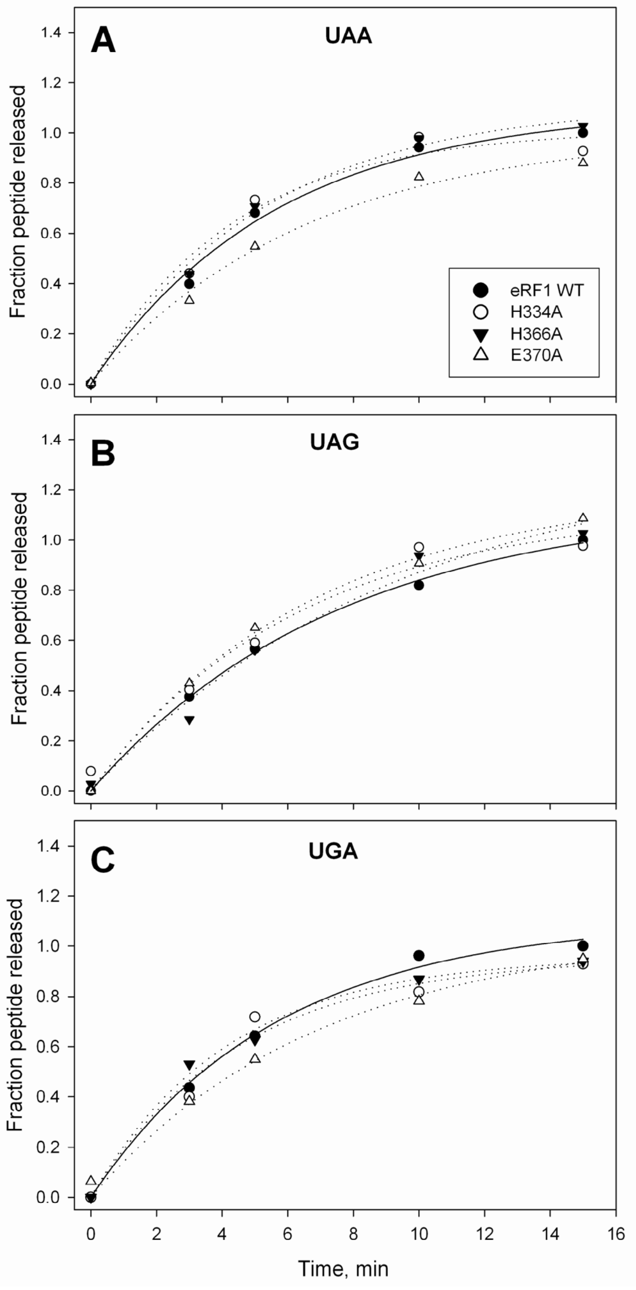

Fig. S8 Results of the gel filtration (A), and SDS-PAGE (8% acrylamide) gel (B) of the C domain of human eRF1.

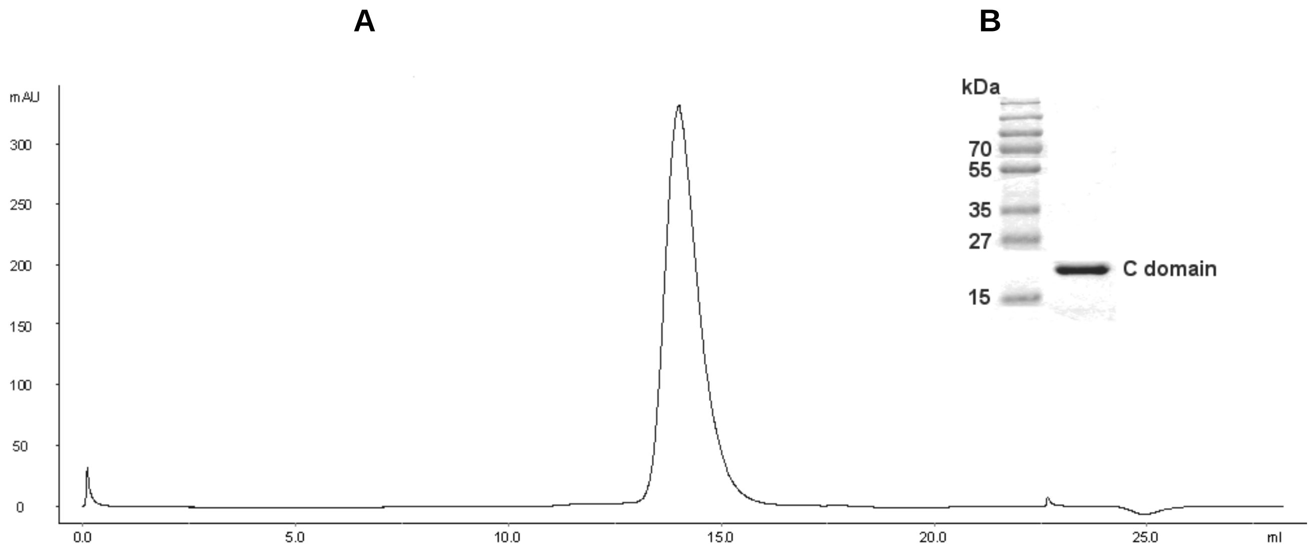

Fig. S9 The stabilization of the loop (residues 358-363) by a network of hydrogen bonds. Residues 358 – 363 of the final 48 calculated structures (of both open and closed conformers) are shown. All structures were superimposed onto the atoms N, Ca, and C' of the residues 359-362 of the representative structure of the open conformer.

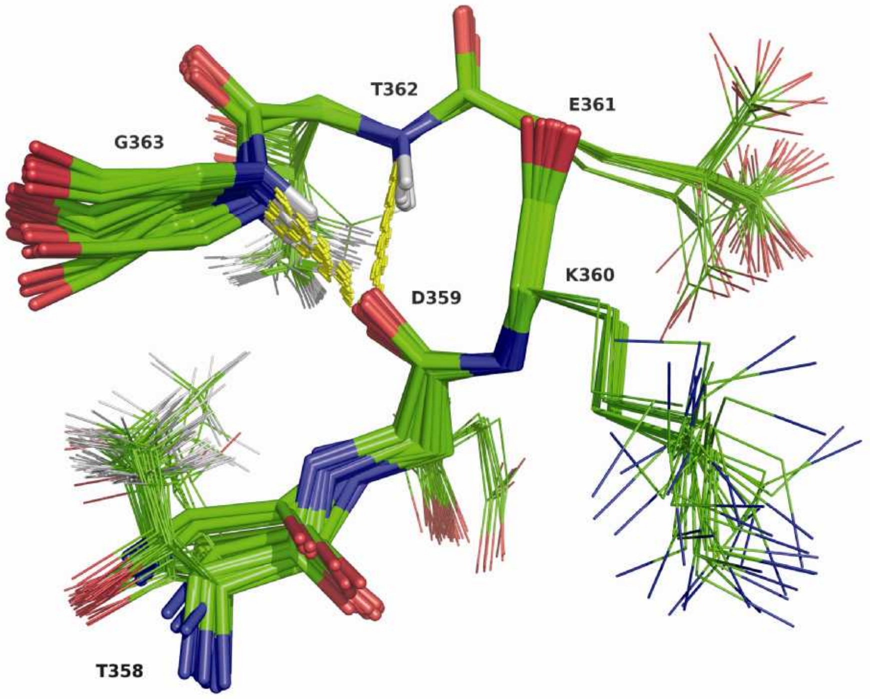

Fig. S10 Multiple sequence alignment of eukaryotic Class-1 polypeptide chain release factors eRF1. Highly and completely conserved residues of eRFs are highlighted in blue/green and red, respectively. The numbering above the sequence corresponds to that of human eRF1.

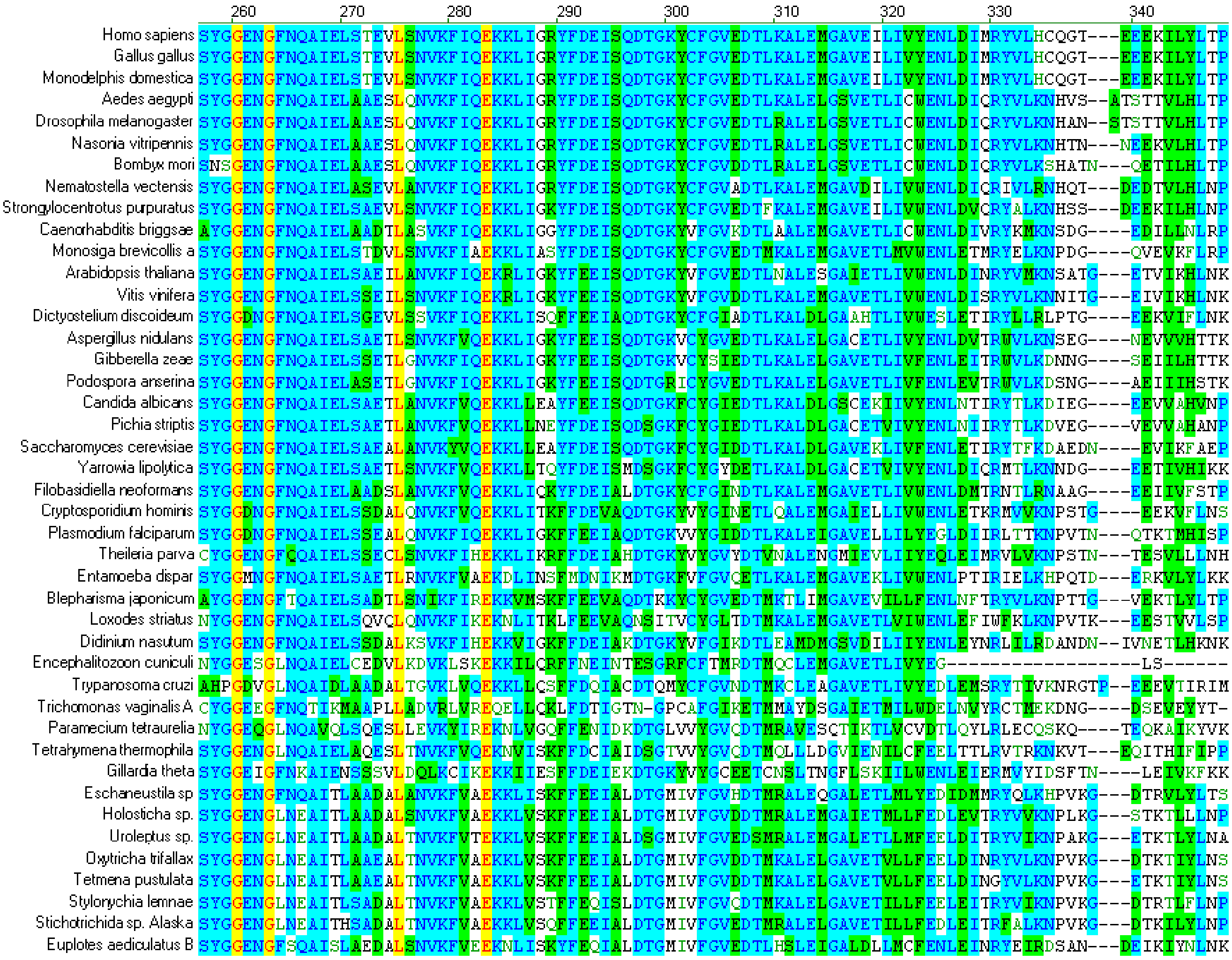

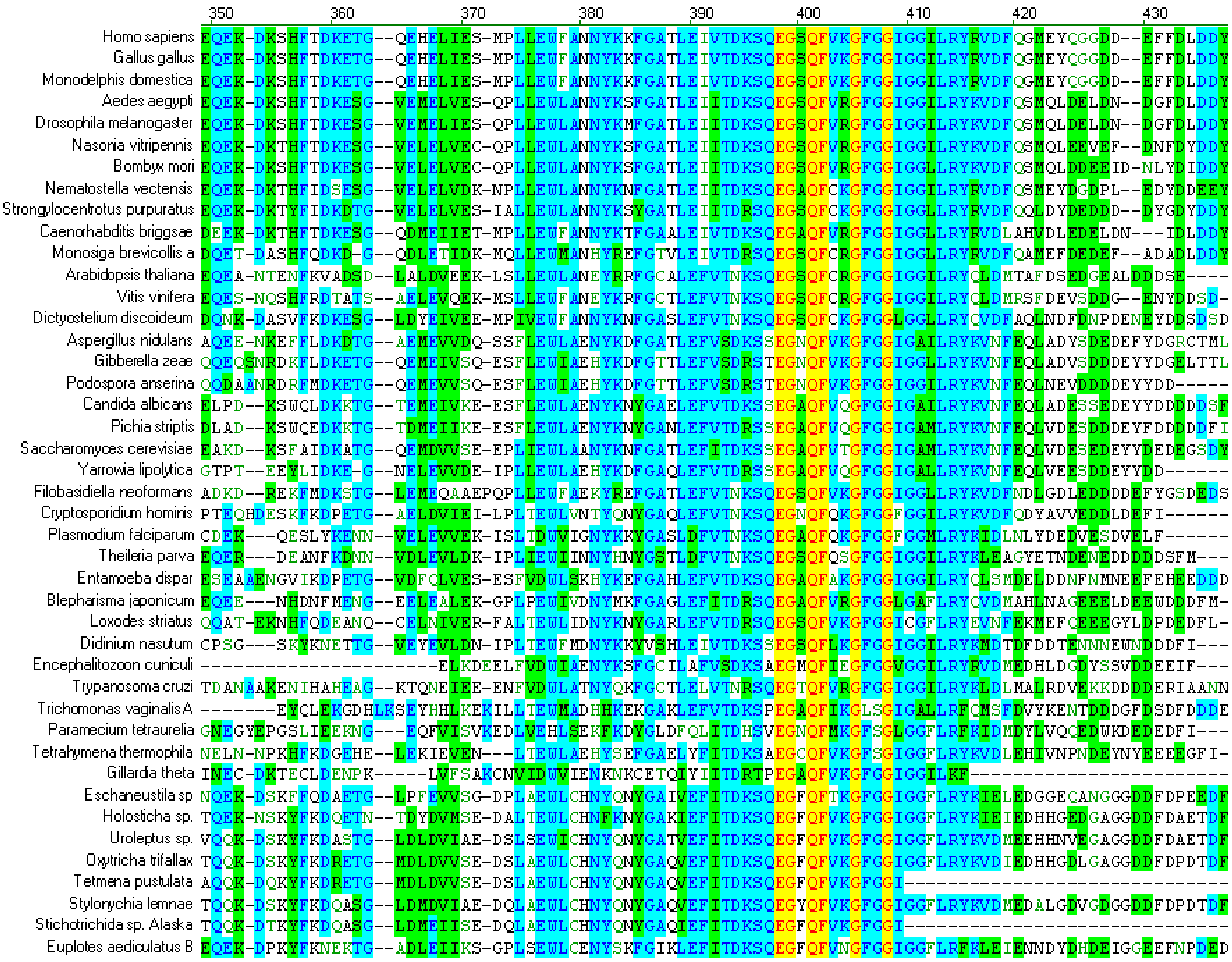
350
360
370
380
390
400
410
420
430
Homo sapiens
Gallus gallus
Monodelphis domestica
Aedes aegypti
Drosophila melanogaster
Nasonia vitripennis
Bombyx mori
Nematostella vectensis
Strongylocentrotus purpuratus
Caenorhabditis briggsae
Monosiga brevicollis a
Arabidopsis thaliana
Vitis vinifera
Dictyostelium discoideum
Aspergillus nidulans
Gibberella zeae
Podospora anserina
Candida albicans
Pichia striptis
Saccharomyces cerevisiae
Yarrowia lipolytica
Filobasidiella neoformans
Cryptosporidium hominis
Plasmodium falciparum
Theileria parva
Entamoeba dispar
Blepharisma japonicum
Loxodes striatus
Didinium nasutum
Encephalitozoon cuniculi
Trypanosoma cruzi
Trichomonas vaginalis A
Paramecium tetraurelia
Tetrahymena thermophila
Gillardia theta
Eschaneustila sp
Holosticha sp.
Uroleptus sp.
Oxytricha trifallax
Tetmena pustulata
Stylonychia lemnae
Stichotrichida sp. Alaska
Euplotes aediculatus B

Fig. S11 The Ramachandran map plot (ϕ and ψ torsion angles for the protein backbone) of all 24 conformers of the NMR families of solution structures of the closed (A) and open (B) conformers of the C-domain of human eRF1. Glycine residues are marked by triangles and all the other residues are shown as squares.

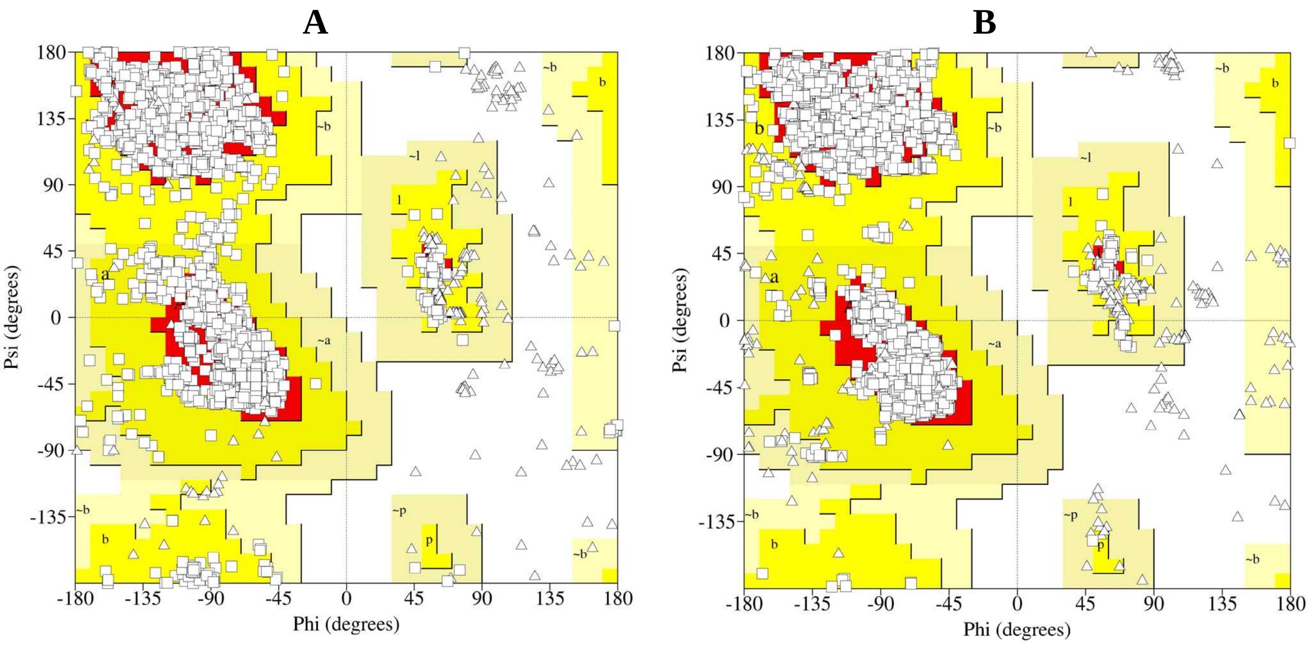

Supplement: Supplementary file 1 [file ejb0277-2611-SD1.pdf]
